# To keep or not to keep? Decision making in adolescent pregnancies in Jamestown, Ghana

**DOI:** 10.1371/journal.pone.0221789

**Published:** 2019-09-04

**Authors:** Luchuo Engelbert Bain, Marjolein B. M. Zweekhorst, Mary Amoakoh-Coleman, Seda Muftugil-Yalcin, Abejirinde Ibukun-Oluwa Omolade, Renaud Becquet, Tjard de Cock Buning

**Affiliations:** 1 Athena Institute for Research on Innovation and Communication in Health and Life Sciences, Vrije Universiteit, Amsterdam, The Netherlands; 2 University of Bordeaux, Inserm, Bordeaux Population Health Research Center, Team IDLIC, Bordeaux, France; 3 Noguchi Memorial Institute for Medical Research, University of Ghana, Legon, Accra, Ghana; 4 Independent Researcher, HealthPro Research and Consultancy, Toronto, Canada; Queen’s University at Kingston, CANADA

## Abstract

**Background:**

Jamestown, an urban coastal slum in Accra, Ghana, has one of the highest adolescent pregnancy rates in the country. We sought to understand the decision (to keep or terminate) factors and experiences surrounding adolescent pregnancies.

**Methods:**

Thirty semi-structured indepth interviews were carried out among adolescents (aged 13–19 years) who had been pregnant at least once. Half of these were adolescent mothers and the other half had at least one past experience of induced abortion. A pretested and validated questionnaire to assess the awareness and use of contraception in adolescent participants was also administered. To aid social contextualization, semi-structured in depth interviews were carried out among 23 purposively selected stakeholders.

**Results:**

The main role players in decision making included family, friends, school teachers and the partner, with pregnant adolescents playing the most prominent role. Adolescents showed a high degree of certainty in deciding to either abort or carry pregnancies to term. Interestingly, religious considerations were rarely taken into account. Although almost all adolescents (96.1%) were aware of contraception, none was using any prior to getting pregnant. Of the 15 adolescents who had had abortion experiences, 13 (87.0%) were carried out under unsafe circumstances. The main barriers to accessing safe abortion services included poor awareness of the fairly liberal nature of the Ghanaian abortion law, stigma, high cost and non-harmonization of safe abortion service fees, negative abortion experiences (death and bleeding), and distrust in the health care providers. Adolescents who chose to continue their pregnancies to term were motivated by personal and sociocultural factors.

**Conclusion:**

Decision-making in adolescent pregnancies is influenced by multiple external factors, many of which are modifiable. Despite legal access to services, options for the safe termination of pregnancy or its prevention are not predominantly taken, resulting in a high number of negative experiences and outcomes. Including safe abortion care within the sexual and reproductive health package, could diminish barriers to safe abortion services. Given the vulnerability of the Jamestown setting, a comprehensive sexual education package that addresses the main decision factors is recommended. Interventions aiming to reduce adolescent pregnancy rates should also recognize that adolescent pregnancies are culturally acceptable in some settings, and under certain circumstances, are desired by the adolescents themselves.

## Introduction

Each year in low and middle income countries (LMICs), approximately 16 million girls aged 15 to 19 years give birth, with 2.5 million of these girls under the age of 16 years [1]. Furthermore, in the period between 2010 and 2014, over 44% of pregnancies reported worldwide were unintended (i.e. unwanted or mistimed) [2]. The burden of unintended pregnancies is discriminately higher in LMICs, and over 56% of these end up in abortions [1]. In Ghana, 25% of women report having had an abortion in their life time [3], and over 11% of maternal deaths are the result of unsafe abortions [2]. Compared to older reproductive age women, adolescents (13–19 years) are more likely to have self-induced abortions contributing substantially to the country’s high burden of maternal mortality and morbidity [4,5]. Adolescent births are also associated with adverse outcomes such as increased risk of caesarian sections, still births, low birth weight babies and neonatal deaths [6–8]. Eliason et al reported an unintended pregnancy rate of 70% in a Ghanaian hospital-based study, with over 90% of adolescent pregnancies being unintended [7].

The confirmation of a pregnancy is accompanied by a decision to abort or continue to term. This decision is influenced by the feelings of the pregnant woman towards the pregnancy, socio-cultural norms and expectations as well as social networks including family and friends [7, 9]. Given the vulnerability of their situation, pregnant adolescents are more likely to struggle with coming to a decision regarding unintended pregnancies. Previous studies have identified mothers of adolescents as the most highly involved actors in the decision making [9–13]. The implication of male partners in the decision making process has also been widely reported in the literature [14–16]. Schwandt et al for example, report that in Ghana, male involvement in the decision making process of unintended pregnancies is in the form of authoritative “orders”, highlighting the role of gender inequality [9]. Bankole et al on the other hand highlight how male denial to assume responsibility of the pregnant adolescent plays a central role in pushing them to opt for abortions, which are at times unsafe [10]. Minors could also be selective in who they involve in their decision making especially where there is history of family violence and a desire to maintain close relationships with their parents [11]. Contrary to what is commonly reported in other LMICs, in Ghana, Geelhoed et al describe women as being sufficiently autonomous in making decisions on how to proceed with an unintended pregnancy [12]. However, their study was not specific to adolescents, being an important vulnerable group.

Understanding the degree of autonomy, and factors influencing decision making among pregnant adolescents are key in carefully identifying their needs, and providing respectful counseling and care. The adolescent health service policy and strategy for Ghana (2016–2020) highlights the need to reinforce early comprehensive sexuality education among adolescents. For instance, one–fifth of women of reproductive age give birth before the age of 18, high awareness rates of modern contraceptive methods are not matched with use, and abortion related stigma remains high despite the relatively liberal nature of the Ghanaian abortion law [17]. Although there have been some studies on sexual and reproductive health decision-making in Ghanaian adolescents, the focus is predominantly on family planning [18,19]. Not many studies have focused on adolescent girls’ decision making experience with pregnancy in Ghana. To address this gap and given the national and global importance of adolescent health, a qualitative study was carried out among 30 female adolescents in Jamestown, Accra, who had either continued a pregnancy to term, or had past experience of at least one self-induced abortion. We sought to understand the adolescent decision making process and outcome, as well as perceived risk factors for adolescent pregnancies. To complement adolescent views, additional interviews were conducted with purposively selected stakeholders (n = 23). Findings would be important for informing the provision of adolescent-centered support in the decision making process and to prevent future unintended adolescent pregnancies.

## Methods

### Study context

Accra is the most populated city in Ghana with about 2.27 million inhabitants [20]. Jamestown is located within the Asheidu Keteke Sub-Metro in the Greater Accra Region, the smallest and most populated Sub-Metro in the Accra Metropolis. Within Asheidu Keteke Sub-Metro, Jamestown is the smallest, yet the most populated and socioeconomically deprived community. It has one of the lowest literacy rates within Accra metropolis [16,18], with the females being the most affected. The community relies primarily on fishing and is populated by a mixture of the indigenous Ga community and migrant groups from various parts of the country. Jamestown is recognised as an important site of the Ga cultural heritage and has a tourist appeal due to its colonial past.

### Data collection

Data were collected through semi-structured interviews with female adolescents who had at least one teenage pregnancy (n = 15) or at least one abortion (n = 15) and 23 stakeholders including health workers and youth activists. Adolescents were asked to discuss the circumstances surrounding their pregnancies, those who informed their decision to terminate or keep the pregnancy, the decision making experience and factors considered in the process. The interviews also explored their role in making the final decision and if they had any regrets. Using probing questions, adolescents were encouraged to recap their decision making experience from confirmation till the final decision to either continue or abort the pregnancy. In addition, a 12-item questionnaire assessing the awareness and use of contraception was administered to each adolescent respondent by trained interviewers (Supplementary file 1). The questionnaire was pretested with a sample of 10 adolescent mothers from the Ussher Polyclinic on their routine medical visits- the main public health facility in Jamestown. Stakeholder respondents were purposively selected based on relationship to pregnant adolescents, frequency of contact with adolescents in health facilities or the community, and involvement in adolescent health promotion programs. These included: a gynecologist-obstetrician, nurses (n = 5), midwives (n = 3), lawyers, (n = 3) teachers (n = 3), a project manager at Ipas (an NGO working on reproductive health and safe abortion care), a project manager for Planned Parenthood Association of Ghana, and youth activists/community mobilizers (n = 3). Additionally, three expert interviews were conducted with experienced general practitioners (n = 2), and a social scientist from Population Council, Ghana who has 15 years experience in adolescent reproductive health. The open-ended questions with stakeholders explored their perceived prevalence of adolescent pregnancies, risk factors, decision making influences and views on adolescent pregnancy and abortion. Due to the stigma associated with abortions, the targeted number of participants was reached through snowballing, starting with the initial group of teenage adolescents who consented to participate in the study. These were recruited at the Ussher Polyclinic with the help of the local youth animator and the head of adolescent health service. Interviews were conducted at the youth-friendly theatre centre in Jamestown, and were conducted in English (by the first author) or Ga (by a trained female local research assistant) languages. All interviews were digitally recorded following informed consent, transcribed, de-identified and validated by an experienced qualitative researcher. Informed by emerging themes and discussions within the research team, research questions were iteratively refined as interviews were conducted. LEB conducted the interviews with stakeholders. LEB, SMY, MBZ, MAC, TCB were involved in the creation of the qualitative coding frame.

### Data analysis

A common coding frame was developed in ATLAS.ti^**©**^ 8 for Windows using open coding and by discussing emerging themes and sub-themes within the research team. This was followed by axial coding to establish meaningful connections between themes using inductive and deductive thinking. Two researchers independently coded (LEB, SMY) the transcripts and met regularly to resolve discrepancies. This helped to minimize bias and improve validity. SMY, RB, TCB, MBZ, LEB, AIO were involved in the iterative data analysis process.

### Ethics

Ethical approval was obtained from the Ethics Review Committee of the Ghana Health Service (GHS–ERC: 003/07/17). The study protocol was also assessed and registered by the Scientific Quality Committee of the Vrije Universiteit Amsterdam-Netherlands (EMGO+; WC2017-025). Prior to registering their consent, all respondents were informed of study aims, measures taken for data privacy and confidentiality, as well as their rights as participants. Participants either signed a consent form or, in the case of minors, assent was obtained.

## Results

### Profile of respondents

The majority (94%) of all respondents (N = 53) were Christians. Adolescents (n = 30) were mainly single (27/30) and not formally employed (17/30) and ranged in age from 14–19years (Mean: 17.8 ±1.5 years). Six adolescents ran small businesses, four were learning a trade while three identified as students. Their ages at first sexual encounter ranged from 10–18 years (Mean: 14.7 ±2.4 years) with 1–3 past unintended pregnancies reported. Thirteen of the adolescents came from single parent households, five from divorced homes, while the others had parents in stable marriages (n = 6) or were orphans (n = 6).

### Thematic overview of results of the self reported own decision process by female adolescents

Three main themes emerged from data analysis: i) The decision making (with two subthemes: Influencers and the Dilemma to keep or not to keep); ii) The abortion experience (with subthemes: Lived experience and Regrets) and; iii) Risk factors for adolescent pregnancies (with one sub-theme- Underlying factors). These are summarized in Table 1 and further discussed subsequently in the narrative, supported by quotes (Table 1).

**Table 1:** Thematic overview of results.

Decision-making	Abortion experience	Risk factors for adolescent pregnancy
**Influencers** *Parents* *Peers and Friends* **Dilemma: to keep or not to keep** *Partners’ responsibility* *Financial autonomy* *Attitude to adoption as an alternative*	**Lived experience** *Negative experiences* *Barriers to safe abortion care* **Regrets** *Reflections and wishe*s	**Underlying factors** *Modern contraception (knowledge*, *availability*, *affordability*, *usage patterns)* *Socio-economic factors* *Community perceptions of early childbearing* *Inadequate comprehensive sexuality education*

### Theme 1: Decision-making

This theme had two main sub-themes: those who influenced the decision making process and how the adolescent dilemma to keep or not to keep was handled. Main factors under this theme are presented in Table 2.

**Table 2:** Decision-making in adolescent pregnancy.

Subtheme/Factor	Supporting Quote
**Influencers**	
-Parents	*“My dad told me he would disown me if I don’t get an abortion”* [Adolescent with an abortion experience #8, 17 years old]
-Peers & Friends	*“I made my friends aware of the fact that I was going to abort it*, *they mocked at me*, *but I wasn’t bothered”* [Adolescent with an abortion experience #4, 17 years old]
**Dilemma: to keep or not to keep**	
-Financial autonomy	*“I am not working*, *and I do not have the money to take care of myself*, *not to talk of taking care of a baby”* [Adolescent with an abortion experience #3, 17 years old]
-Partners’ responsibility	*“I was compelled to go in for an abortion because the guy who impregnated me ran away”* [Adolescent with abortion history #10, 18 years old]
-Abortions (knowledge & attitude)	*“Many girls die from abortions in Ga communities”* [Adolescent mother#9, 18 years old]

### Influencers in decision making

Interviews revealed that the main influencers in the decision-making process were: parents (mothers and fathers), friends and family members of adolescents. Fathers influenced the process in a top-down manner- commanding their adolescent daughters on a specific course of action. This influence included threats of being disowned if the pregnancy was not terminated as keeping it would bring shame to the family.

*“My dad told me he would disown me if I don’t get an abortion*”

[Adolescent with an abortion experience #8, 17 years old].

Others who opted for abortions were also influenced by lack of financial or moral support from their partners, and their desire to continue schooling or to learn a trade, which they felt the pregnancy and a baby would hinder.

Majority of adolescents who continued their pregnancies to term reported that their mothers played a key supportive role in the decision. Peer opinion did not seem to discourage adolescents who opted for abortions. Despite sometimes opposing views from external influencers, most adolescents felt the final decision was an individual one, emphasizing their autonomy.

“*During the pregnancy, my mother and siblings asked me to go in for an abortion. I had to leave home to stay with my friend’s mother till I gave birth*”

[Adolescent mother #4; 17 years old].

All stakeholder respondents believed the family should play a significant role in the decision-making process. However, views diverged on who should be responsible for the final decision. Health care personnel leaned towards adolescent autonomy in final decision making.

*“Oh definitely*! *I mean when it comes to age and especially when people are below the ages of eighteen*, *you are considered a minor*. *You would definitely need the help of another person*. *But then again*, *the final decision should come from you*”

[Nurse #4].

Other interviewed stakeholders on the other hand, including lawyers, felt adolescents were immature, inexperienced and not capable of making rational decisions. This group opined that parents, especially mothers, knew exactly what was best for their children.

Pressure from parents pushed two adolescents into abortions against their will. One parent threatened to disown the child, while the other cited the avoidance of shame.

Not being able to recognize the early signs of pregnancy meant some adolescents were left with no choice but to continue their pregnancies to term due to gestational limits.

*“I wanted to have an abortion*. *When I went there*, *they asked me to go for a scan*. *The scan revealed that I was 7 months*, *3 days pregnant*. *I decided to keep the pregnancy because the baby had already turned into a human being*”

[Adolescent mother#4, 18 year old].

### Dilemma: To keep or not to keep

**Fig 1:**
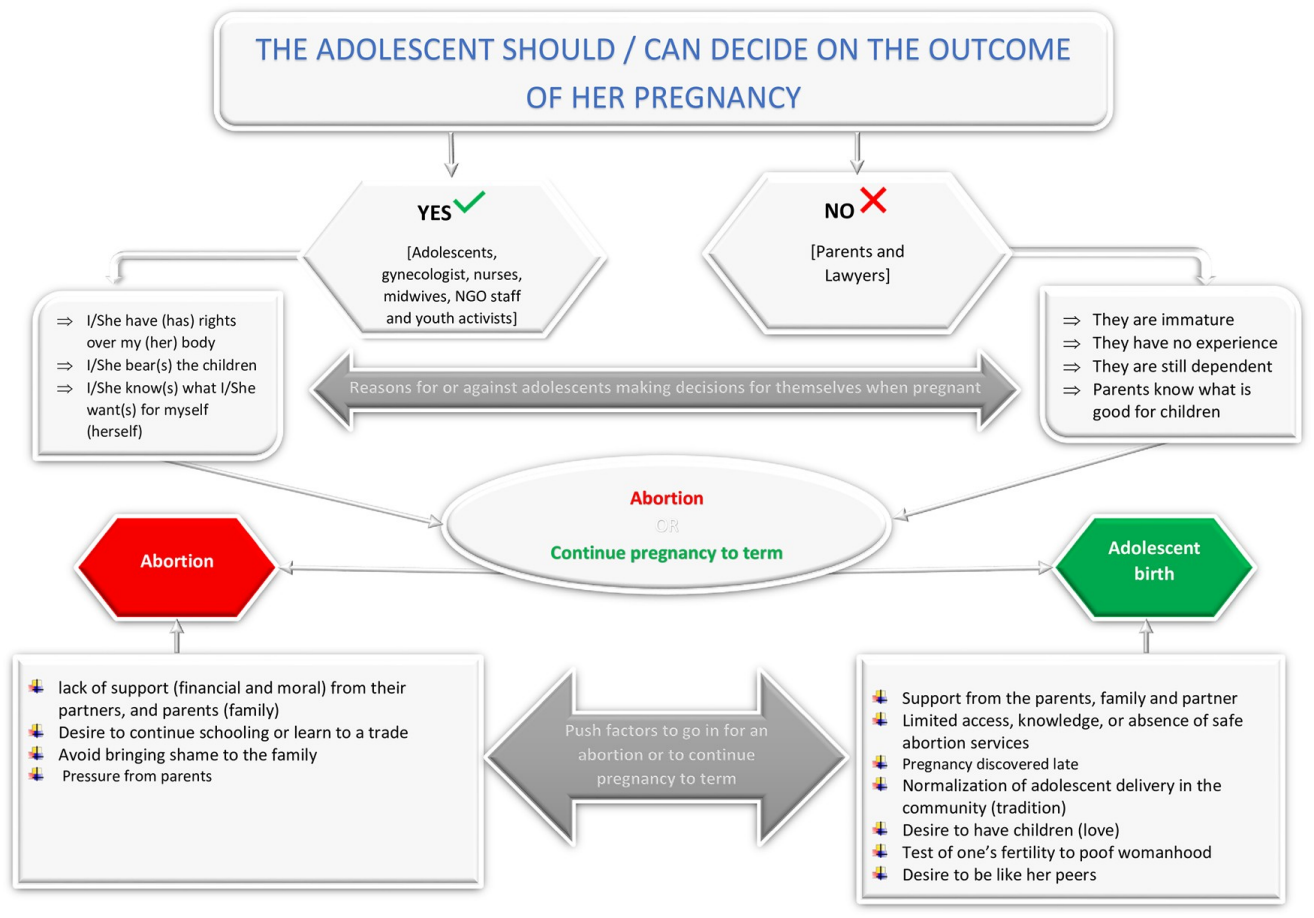
Adolescent pregnancy decision-making in Jamestown, Ghana.

Fig 1 summarizes the decision making pathways to keep or not to keep an adolescent pregnancy in Jamestown. Adolescents reported multiple factors they considered in navigating the dilemma of choice- to either continue the pregnancy to term or induce an abortion. In addition to the role of influencers earlier described, other factors included individual dispositions and community norms and perceptions towards adolescent pregnancies, abortion and adoption. In addition, health system factors which influence the availability and access to safe abortion services and modern contraceptives mediated this dilemma.

Community clinic nurses felt that adolescents give birth to assert their fertility. Affirming this were reports from some adolescents who stated their desire for children compelled them to carry on with their pregnancies.

“*Here we are talking about the Ga community; Jamestown, Chorkor, Korlikornor, Korle-bu. The act of pregnancy in itself is a test of fertility, and so once you see your friend with a child, you also want to be sure*”

[Ipas project coordinator]

One adolescent mother was motivated to carry the pregnancy to term in order to continue her family lineage.

“*I am the only child to my mother, so I had to give birth to continue our lineage*”

[Adolescent mother #11, 18 years old]

Availability of adequate finances was the main factor taken into consideration in resolving the dilemma for most adolescents who opted for abortions. This was less of a concern for adolescents whose partners accepted to take responsibility for the pregnancy.

While a couple of teenagers were forced to abort against their will, some adolescents said they have would have preferred to abort but did not due to negative views held about abortions, and having heard sad stories on the aftermath of an abortion. Most were scared of dying following the procedure- a common outcome in the community. Furthermore, lack of awareness on existing legislation was a barrier to accessing available safe abortion services.

“*They know that some form of abortion law is exists, but the details of it they don’t know. Most people don’t know that almost all pregnancies qualify for legal safe abortions*…”

[Gynecologist]

Interviews also explored respondents’ views on giving up babies for adoption as an alternative to unsafe abortions which was the route many teenagers took. Surprisingly, both adolescents and the wider group of stakeholders expressed negative views on adoption.

“*Adoption is not good, its better to go in for an abortion if you cannot cater for the child*”

[Adolescent mother #8, 18 years old]

“*No, I hate to hear that. I think everyone should take responsibility of her own children*”

[Adolescent mother #2, 16 years old]

Interestingly, although Jamestown is a very religious community, religious beliefs were not mentioned by adolescents as a motivation to continue pregnancies to term.

### Theme 2: Abortion experience

Fifteen adolescents had at least one experience of an abortion and interviews investigated their abortion experience, revealing two main sub-themes- lived experience of abortion and regrets following the abortion.

### Lived experience

Most respondents in this group reported very negative abortion experiences. These ranged from intense pain, excessive bleeding and post-abortion hospitalization.

“*I felt very sick and almost lost my life*”

[Adolescent with an abortion experience#4, 18 years old]

“*No one knew about it till I fainted. It was not until I was taken to the hospital that people came to know that I had an abortion*”

[Adolescent with an abortion experience # 7, 17 years old]

Only two of the 15 adolescents who had an abortion used a qualified health care professional. One girl still had post-abortum complications requiring hospitalization. Others who self-induced (n = 13) their abortions used unspecified herbal medicines, tablets, and alcoholic concoctions either purchased at a community store or prepared at home.

“*Yes, they have a lot of concoctions. Some grind glass bottles to drink […] some take mixtures of tablets, drink Guinness (alcohol). Others lie down for people to jump on them, while others insert roots and sticks into the vagina to force open the cervix. They have a lot of methods and concoctions*…”

[Nurse #1, Unit head Adolescent clinic]

The gynecologist confirmed that incomplete abortions were one of the main reasons for gynecological emergencies.

“*We see a lot of pregnancies at the gynae unit that have attempted unsafe abortions before, and then they come because they were not successful, or developed some complications*”.

[Gynecologist]

Summarily we identified four main factors that hinder access to safe abortion care in Jamestown:

1. Confidentiality and privacy issues: Most adolescents were generally afraid that health care providers might inform their relatives or community members.
2. Safe abortions are unaffordable for the majority- costing between GHS 50–750 ($11 –$165), depending on the health facility and gestational age.
3. Most adolescents including those eligible are unaware of their legal access to safe abortion services.
4. Culture of fear: communities generally associated abortions with grave complications.

### Regrets

Most teenage mothers with an abortion experience maintained they had no regrets about their decision, and would make the same choice in similar future circumstances. The exceptions included those who had been pressured by their parents to abort. While a few expressed regret, it paled in comparison to the opportunities they would have lost in the event of continuing the pregnancy. Regret was sometimes due to the complication from the unsafe procedure.

“*No*, *I felt very bad*, *but did it because I wanted to go back to school”*

[Adolescent abortion group #6, 17 years old]

“*Yes I regretted, because I started bleeding again and had to be hospitalized for two more days*”

[Adolescent abortion group #7, 16 years old]

### Theme 3: Risk factors for adolescent pregnancies

Numerous predisposing factors for adolescent pregnancies were identified: sub-optimal use of modern contraceptive methods, socio-economic factors, inadequate comprehensive sexuality education and community normalization of early adolescent pregnancies. The main factor was related to sub-optimal use of contraception. Results of the 12-item questionnaire (S1 Table) assessing the awareness and use of contraception is presented in Table 3.

**Table 3:** Awareness and use of contraception.

Question	Response	n (%)
Have you ever heard of contraception?	Yes	29 (96.7%)
	No	1 (3.3%)
Have you ever used any form of contraception before?	Yes	13 (43.3%)
	No	17 (56.7%)
Are you currently using any form of contraception?	Yes	7 (23.3%)
	No	23 (76.7%)
Were you using any form of contraception before getting pregnant?	Yes	5 (16.6%)
	No	25 (83.4%)
From where did you learn of contraception?	Friends	6 (20.0%)
	Family	1 (3.3%)
	Health care provider/ health talk	23 (76.7%)
Have you heard of emergency contraception before?	Yes	14 (46.6%)
	No	16 (53.4%)
Have you used emergency contraception before?	Yes	4 (13.3%)
	No	26 (86.7%)

Only five of the 30 adolescents interviewed were using some form of contraception prior to becoming pregnant. However, almost all of them were aware of contraceptive methods but over half had never used any form of contraception.

Some adolescents reported that they intentionally decided to get pregnant and deliberately refused to use any form of contraception.

“*I just decided not to use condom, I wanted to be pregnant and give birth*”

[Teenage mother #7; 17 years old].

While some adolescents did not use contraception by choice others reported they would have liked to use condoms but did not because their partners’ disliked them.

The main barriers to contraception uptake were: false beliefs, poor message framing by health personnel, privacy and confidentiality concerns, fear of side effects (dizziness, weight gain, pain), potential stigma at point of purchase. Interestingly, there was indication of a higher sense of stigma and myth surrounding condoms compared to other contraceptive methods. The cost of contraceptives was not reported as a barrier to uptake.

“*I heard people saying it in the public transport that it is not good to use the condom because it will end up making you sick*”

[Adolescent mother 3]

*“The way we approach education and communication on contraception is a big problem*. *We focus more on talking of family planning for which reason they think that they don’t have a family*, *and so it does not concern them*.”

[Ipas project coordinator]

Identified underlying factors are briefly reported below:

### Normalization of early adolescent pregnancies

In Jamestown, adolescent pregnancies were viewed by both parents and adolescents as a normal occurrence in the community, irrespective of marital status. As one community nurse noted, it was not considered normal for females above 20 years to not have children- concerns that could lead to rumors and stigmatization.

“*Oh you know the tradition here. If you are fifteen sixteen years and you are not pregnant, they think you are not normal. So when you find a female who is twenty one years, twenty four and not pregnant or having any children, the community will be mocking at the person*.”

[Nurse #5]

In addition to the cultural and societal pressure within Jamestown, a few stakeholders also cited parental pressure on their teenage girls to have grandchildren as soon as possible.

“*I think there’s a lot of pressure from parents on young people, particularly young girls to engage into sexual activity […]. We’ve heard many young people say they have been questioned by their parents if they do not see their friends who have children already […] not in a manner that reflects an aspiration to become a better person or whom you want to be, but an aspiration to be like other people*.”

[Youth activist, 2]

### Poverty, illiteracy and unemployment

Low literacy rates among young adults in the area was considered a major contributing factor to the high rates of unintended pregnancy by all stakeholders. A few parents (n = 3) however, attributed the high adolescent pregnancy rates to lack of parental care. Engagement in the informal sector in order to meet financial obligations resulted in many adolescent girls being out of school. Jamestown is also one of the poorest urban slums in Ghana and very high unemployment rates and poverty drives the local sex industry and prostitution. Some respondents noted that parents could push their daughters into prostitution in order to support their families financially. Some girls were reported to strategically get pregnant for men with higher economic means in order to benefit financially through child support.

### Insufficient comprehensive sexuality education

Sex education at home and in schools was considered absent, suboptimal or inappropriate. Parents reportedly shy away from discussing sexuality issues with their wards. Some considered that the children were too young, or that the information could instead tarnish their moral compass. For instance, some parents thought that discussing issues regarding use of contraceptives would push adolescents into having sex.

“*That’s all. Abstinence. You are an adolescent, what are you doing with sex? You should be in school studying and thinking about your future and doing something else. If you are going around sleeping around…if you allow or offer them contraceptives, then you are giving them the leeway to go about having sex and getting pregnant, and putting toxins in their systems*.”

[Lawyer #2]

The current curricula for sex education was considered inadequate or infective by some stakeholders, especially the dominant messaging on abstinence. Sex education was further described as commencing late or only carried out to ensure secondary school students pass their examinations.

“*This message has been preached for forty years and over, but more and more adolescents still continue to get pregnant. It simply means it does not work*”

[Ipas project coordinator].

## Discussion

In this study, we sought to understand the decision-making experience in adolescent pregnancies and considerations in either continuing a pregnancy to term or opting for an abortion. We further explored the risk factors of adolescents’ pregnancies in Jamestown from the perspectives of adolescents and key stakeholders.

### Autonomy versus influence

Findings showed that despite prevailing influence from parents and peers, there was a high degree of certainty among adolescents in deciding to either abort or carry pregnancies to term. This was however not always the case; specific instances showed that authoritative decision making overruled autonomy or shared-decision making, especially where fathers were the main influencers. We found differing views on the extent to which parents should influence the final decision for pregnant adolescents, although all stakeholders agreed there should be some level of supportive parental involvement in decision—making. Adolescents’ autonomy in deciding to abort or keep a the pregnancy was therefore modified by the extent to which they are dependent on family support, the extent to which other third parties like mothers can mediate the decision outcome, and the general influence of power within the family and community setting [15,16]. Mothers might be more understanding towards pregnant adolescents because of their maternal disposition to protect and nurture, or because they were pregnant at a similar age, and can therefore relate to the experience of their daughters. In a case–control study to measure pregnancy decision resolution among 1,324 pregnant Australian teenage mothers, teenagers whose mothers had become pregnant as teenagers had reduced odds (0.4) of recommending an abortion for their daughters [21]. A few studies in similar contexts have emphasized the role of the family as central in the decision making process [9,13,16,22], although this could be more the case in sub-Saharan Africa than in Western countries where freedom and autonomy is a given even at a young age [23]

Although partner responsibility was a key factor in either keeping pregnancy to term or opting for an abortion, no adolescent reported that their final decision was made by the partner. Contrary to our findings, Schwandt et al. in a qualitative study in two Ghanaian referral hospitals reported that partners directly influenced pregnancy decisions through “orders”, especially in the case of abortions [9]. Irrespective of the fact that most of the respondents (95%) were Christians, religious considerations were not taken into account in the decision making process. Adolescents who chose to continue their pregnancies to term are motivated by personal, economic, and sociocultural factors like continuing the family lineage, proving their fertility, and at times to benefit from financial support from the partner [18, 24].

### When the decision is not to keep

Our study identified multiple reasons why adolescents chose abortion over carrying pregnancies to term. These included partner denial of responsibility, financial and study or employment considerations, and parental pressure. Most of the adolescents did not express regret about their pregnancy decisions, but we could not identify studies from Ghana or other African countries that reported decision certainty in adolescent pregnancies. Indeed, some authors have reported that women who opted for abortions generally did not change their minds, even after pre-abortion counseling sessions [25,26]. These studies were however not carried out among adolescents. It is possible that some pregnant adolescents who reported taking the final decision to continue the pregnancy to term by themselves, would have initially wanted an abortion, especially for those whose pregnancies were discovered late. Retrospectively evaluating their decisions is a possible source of bias. Joyce and colleagues have highlighted the fact that retrospective determination of pregnancy intention is likely to be influenced by the presence of the infant- the smile of the child could lead to a more positive recollection of the past [27].

Beyond the limits imposed by gestational barriers, it was clear from our findings that although respondents were generally aware of abortion services in Ghana, there was a lack of awareness on the extent to which the existing legislation was applicable to them. This gap was a major barrier to accessing available safe abortion services resulting in unsafe practices which have been known to endanger adolescent lives [28,29]. Of the 15 adolescents with an abortion experience, 87.0% were carried out under unsafe circumstances. Although the abortion law in Ghana is one of the most liberal in Africa [30], abortion stigma and ignorance regarding the law remain a main hindrance to access to safe abortion services, affecting both clients and service providers [9, 29, 31]. The Ghanaian abortion law of 1985 permits abortion in cases of rape, incest or the “defilement of a female idiot;” if the life or health of the woman is in danger; or if there is risk of fetal abnormality [32]. The law permits abortions for mental reasons. However, a mental state assessment by a psychiatrist is not required. Hanschmidt et al in a systematic review of six countries, reported fear from social judgement, self-judgement and secrecy, among women who had abortions [31]. In the Ghanaian setting, a study found three main ways whereby abortion stigma can be perpetuated: lack of overt institutional support regarding safe abortion care, negative provider attitudes towards women seeking abortion services, and religious beliefs against abortions [29, 33]. It is therefore important to increase awareness campaigns regarding the possibility of obtaining abortions on request without fear of prosecution, both for the public and health care staff.

Financial considerations were also identified as a barrier to accessing safe abortion services. The Ghana Health Service- the authority mandated to regulate health service delivery and practices—is yet to establish guidelines to unify the cost of safe abortion services. The Reproductive Health Strategic Plan (RHSP) stated the national health strategy for reproductive health in Ghana over a five year period from 2007 to 2011 [34]. Ensuring the availability of comprehensive abortion care (CAC) services as permitted by law was part of this strategic plan, within the first strategic objective, aiming at reducing maternal morbidity and mortality. Distrust in health service providers has been reported as a main barrier that limits access to safe abortion services in Ghana [18,33] Distrust in health care staff is a major contributor to repeat adolescent pregnancies and unsafe abortions [18,33]. Challa et al in Ghana reported limited access to confidential sexual and reproductive health services as a main detractor for adolescents to seek care in health facilities. Some adolescents who sought contraceptive services were considered as “bad girls 18]. Other studies have reported negative health care provider attitudes in Ghana. For instance, Schwandt et al. highlighted how the harsh attitudes of nurses towards safe abortion care could affect the quality of post-abortion services, as well as deter clients from seeking care [9]. Oppong-Darko et al (2017) in a study among Ghanaian midwives highlighted how religious convictions deter some providers from providing abortion services [28]. In a qualitative study involving 43 health care providers across different levels of management (managers, obstetricians, midwives) in three hospitals in Accra, religious views were identified as key potential barriers in the provision of safe abortion care. Midwives, for instance, were most likely to condemn abortions as sinful [29]. If abortion care were incorporated into the reproductive health policy and package of the MoH, it could reduce the stigma that affects both abortion providers and users. It is important that the safe abortion care policy ensures that conscientious objection from service providers does not affect service provision.

Surprisingly, both adolescents and the wider group of stakeholders expressed negative views on adoption, with some adolescents preferring to abort or to continue pregnancies to term. This echoes findings in a United States study where abortion was preferred, and only 9% of those who were denied abortions (161 women) due to gestational limits, put their children up for adoption post-delivery [35]. There are not many studies on adoption as an alternative decision in teenage pregnancies, but there is justification to research this further, especially where the abortion option as in our case resulted in mostly unsafe and life-threatening procedures.

### The abortion experience

Abortions are not uncommon in Ghana; over 25% of women in Ghana have had at least one abortion in their lifetime [36]. Adolescents in our study who underwent unsafe abortions (13/15) reported distressful experiences from complications such as persistent bleeding and hospitalization. Respondents reported many cases of abortion-related deaths within the Jamestown community. Unsafe abortions are a major contributor to maternal deaths in Ghana accounting for 11.0% of maternal deaths [36], with adolescents being the most affected group [24,36]. Since over 56% of unintended pregnancies will end up as abortions [1], it is of interest to step up the availability, accessibility and use of safe abortion services within this community.

### Truly unintended?

The cultural desirability to have children as early as possible to prove one’s fertility or continue the family lineage, as well as parental pressure to become grandparents played significant roles in pushing adolescents to either get pregnant, or to continue pregnancies to term once pregnant. Some adolescents were also found to intentionally get pregnant for financial benefits. In a qualitative study on the experiences of pregnancy and motherhood among teenage mothers in the Ga East Municipality, a suburb of Accra, a few adolescents intentionally got pregnant to command respect from the society [37]. It is therefore erroneous to assume that all adolescent pregnancies in this setting are unintended. Interventions to prevent adolescent pregnancies should therefore be more inclusive—targeting both unintended and intended pregnancies [37]. Gordon recommends that strategies to reduce intended adolescent pregnancies should focus on helping adolescents embrace alternatives to pregnancy, such as educational pursuit or learning a trade, while those targeting unintended pregnancies should promote knowledge on modern contraceptive use and overcome barriers to their uptake [38].

### Risk factors for adolescent pregnancies

Although almost all adolescents (96.1%) we interviewed were aware of contraception, none was using any prior to getting pregnant. In our study, the high level of awareness regarding modern contraceptive methods was unfortunately matched by a very low usage rate. A respondent attributed this to poor program messaging- where the terms ‘contraception’ and ‘family planning’ have been used interchangeably, resulting in adolescents distancing themselves from the need for family planning and by extension, contraception. Low uptake, despite being readily available, affordable and cheap is an issue of concern and sheds light on gaps in the national family planning strategy. Male refusal to use any form of contraception like condoms, being unperturbed if the adolescent becomes pregnant or not, and potential side effects from hormonal contraception were the main predictors of non-use of contraception in a representative survey of 18-24year olds in Accra [39]. Abdul-Rahman et al after analyzing the 2003 and 2008 demographic health survey data of Ghana highlight cost and misconceptions about the effects of contraception among 15–19 year old females [40]. In qualitative study in Iran among 13–19 year old married teenage mothers, being unfamiliar with contraceptive methods, pressure to become pregnant and misconceptions were the main factors that influenced contraceptive prevalence [41]. While highlighting the role of the social context in young women’s contraceptive decisions making, Mutumba et al (2018) in a multi country analysis highlight the fact that an increase in community levels of education could improve contraceptive use among adolescents. Gender imbalance in decision making and fertility related norms also adversely influence contraceptive use among young women [42]. Increasing contraceptive uptake is context dependent, and might warrant more qualitative studies.

In Jamestown, adolescents engage in sexual activity from as early as age 10 from our findings. Challa et al. [18] in a study among 15–24 year old women in Kumasi and Accra, Ghana reported the cultural unacceptability of adolescent sexuality education as being detrimental to adolescent reproductive health. Although no instances of sexual violence were reported in this study, Gyesaw and Ankomahs’ findings from the Ga East Municipality of Accra noted cases of sexual violence, and partner coercion including rape [37]. With the acceptance of early child bearing and the taboo surrounding discussion of sexuality issues between parents and children, parents may not be paying enough attention to the circumstances under which their daughters get pregnant. It is therefore possible that cases of sexual coercion and abuse go unnoticed and unaddressed, propelling other social stigmas.

## Other recommendations

With adolescent health as a key agenda in the global strategy for sexual and reproductive health and rights, the importance of informed decision-making cannot be overemphasized in the provision of respectful adolescent care [18]. Early comprehensive sexual and reproductive health education is invaluable in empowering adolescents to prevent unwanted pregnancies, identify early signs of pregnancy in order to initiate decision-making and to act in a timely fashion. This would also prevent late-term abortions and facilitate earlier and safer access to services should they decide to terminate. For those who decide to keep their pregnancies, they can also initiate antenatal care visits on time [20].

Health system barriers to optimal safe abortion care such as cost, information access, trust and confidentiality need to be carefully addressed. Jamestown has an adolescent reproductive health unit within the state owned health facility (i.e. the Ussher Polyclinic). Providing adolescent friendly services incorporated with continuous monitoring and supervision would be welcome. Considering the reported early onset of sexual activity in Jamestown, sexual health education should preferably be initiated in the pre-teenage years, with emphasis on the benefits of delaying initiation of sexual activity and knowledge of contraceptive methods. Health services at the community level that are adolescent friendly in principle and practice are needed to establish trust between service providers and adolescents, who often cannot discuss sensitive issues with parents. Our study further highlights that in the absence of practical information on the legal stipulations of abortion laws, many who need such services might never access them even where they are available.

## Limitations

While this study shed valuable insights on the underlying causes and modifying factors of adolescent pregnancy experience and decision making, our findings cannot be generalized to the Accra metropolis. Accra represents a more diverse blend of cultures and the general attitudes to early childbearing and adolescent sexual health and decision-making are likely different. Additional studies in other Ga communities as well as in other cultural groups and regions, could provide further insight on the overall picture of adolescent pregnancy decision-making in Ghana.

## Conclusion

Decision-making in adolescent pregnancies is influenced by multiple external factors, many of which are modifiable. Adoption as an alternative to unwanted pregnancies does not seem to be a popular option, which narrows decision options (either to keep or not). Without targeted community and facility level interventions aimed at addressing knowledge, trust and financial barriers to accessing preventive and safe abortion options, female adolescents remain at a high risk of mortality from unsafe abortions. Policy wise, including safe abortion care within the sexual and reproductive health package, could diminish barriers to abortion services. A comprehensive sexual education package that addresses the main decision factors identified in this study is a recommended action point in vulnerable settings like Jamestown. Knowledge and awareness efforts should also be initiated in the immediate pre-teenage period. Nevertheless, interventions aiming to reduce adolescent pregnancy rates should also recognize that adolescent pregnancies are culturally acceptable in some settings and under certain circumstances are desired by adolescents themselves.

## Supporting information

S1 Table 12—Item questionnaire used to assess awareness levels and use of modern contraception. (DOCX)
