# Correction: To keep or not to keep? Decision making in adolescent pregnancies in Jamestown, Ghana

**DOI:** 10.1371/journal.pone.0223453

**Published:** 2019-09-30

**Authors:** Luchuo Engelbert Bain, Marjolein B. M. Zweekhorst, Mary Amoakoh-Coleman, Seda Muftugil-Yalcin, Ibukun-Oluwa Omolade Abejirinde, Renaud Becquet, Tjard de Cock Buning

The fifth author's name is ordered incorrectly. The correct name order is: Ibukun-Oluwa Omolade Abejirinde. The correct citation is: Engelbert Bain L, Zweekhorst MBM, Amoakoh-Coleman M, Muftugil-Yalcin S, Abejirinde I-OO, Becquet R, et al. (2019) To keep or not to keep? Decision making in adolescent pregnancies in Jamestown, Ghana. PLoS ONE 14(9): e0221789. https://doi.org/10.1371/journal.pone.0221789
