# Supplementary material for: To keep or not to keep? Decision making in adolescent pregnancies in Jamestown, Ghana
Source: PLoS One. 2019 Sep 4;14(9):e0221789. doi: 10.1371/journal.pone.0221789 (PMC6726415; doi:10.1371/journal.pone.0221789)
Supplement: S1 Table — (DOCX) [file pone.0221789.s001.docx]

**Knowledge, awareness and use of contraception among adolescent mothers in James Town, Accra, Ghana**

1. Have you ever heard of contraception of family planning?
2. Yes
3. No
4. If yes, from where did you learn of this (multiple answers applicable)?
5. Health care provider
6. Television
7. Radio
8. Internet
9. Newspaper
10. School
11. Parents
12. Friends
13. Which types / methods of contraception do you know?

________________________________________________________________________________________________________________________________________________________________________________________________________________________

1. Have you ever used any method of contraception before?
2. Yes
3. No (If No, skip to question 10)
4. If yes, which method (s) of contraception have you ever used?
5. Are you currently using any method?
6. Yes
7. No
8. How regular did/do you use this method?
9. All the time
10. Some time
11. Never
12. If you have used a method of contraception before, did it ever happen that you stop using it, or use it at times?
13. Yes
14. No
15. If you ever used a method inconsistently or stopped, what are the reasons that led to this?

_______________________________________________________________________________________________________________________________________________________________________________________________________________________________________________________________________________________________________________________

1. For those who have heard, but never used any method of contraception before, can you explain why you have never used modern contraception?

1001. Don’t have money

1002. Distance from the health facility

1003. Shame or stigma

1004. Because I heard of the negative side effects of modern contraceptives

1005. Not available at the health centre

1006. Others

1. Have you heard of emergency contraception before?
2. Yes
3. No
4. Have you used emergency contraception before?
5. Yes
6. No
